# Lack of Mucosal Immune Reconstitution during Prolonged Treatment of Acute and Early HIV-1 Infection

**DOI:** 10.1371/journal.pmed.0030484

**Published:** 2006-12-05

**Authors:** Saurabh Mehandru, Michael A Poles, Klara Tenner-Racz, Patrick Jean-Pierre, Victoria Manuelli, Peter Lopez, Anita Shet, Andrea Low, Hiroshi Mohri, Daniel Boden, Paul Racz, Martin Markowitz

**Affiliations:** 1 Aaron Diamond AIDS Research Center, Rockefeller University, New York, New York, United States of America; 2 Department of Medicine, Division of Gastroenterology, New York University School of Medicine, New York, New York, United States of America; 3 Bernhard-Nocht Institut Fur Tropenmedizin, Hamburg, Germany; Tulane University Medical School, United States of America

## Abstract

**Background:**

During acute and early HIV-1 infection (AEI), up to 60% of CD4^+^ T cells in the lamina propria of the lower gastrointestinal (GI) tract are lost as early as 2–4 wk after infection. Reconstitution in the peripheral blood during therapy with highly active antiretroviral therapy (HAART) is well established. However, the extent of immune reconstitution in the GI tract is unknown.

**Methods and Findings:**

Fifty-four AEI patients and 18 uninfected control participants underwent colonic biopsy. Forty of the 54 AEI patients were followed after initiation of antiretroviral therapy (18 were studied longitudinally with sequential biopsies over a 3-y period after beginning HAART, and 22 were studied cross sectionally after 1–7 y of uninterrupted therapy). Lymphocyte subsets, markers of immune activation and memory in the peripheral blood and GI tract were determined by flow cytometry and immunohistochemistry. In situ hybridization was performed in order to identify persistent HIV-1 RNA expression. Of the patients studied, 70% maintained, on average, a 50%–60% depletion of lamina propria lymphocytes despite 1–7 y of HAART. Lymphocytes expressing CCR5 and both CCR5 and CXCR4 were persistently and preferentially depleted. Levels of immune activation in the memory cell population, CD45RO^+^ HLA-DR^+^, returned to levels seen in the uninfected control participants in the peripheral blood, but were elevated in the GI tract of patients with persistent CD4^+^ T cell depletion despite therapy. Rare HIV-1 RNA–expressing cells were detected by in situ hybridization.

**Conclusions:**

Apparently suppressive treatment with HAART during acute and early infection does not lead to complete immune reconstitution in the GI mucosa in the majority of patients studied, despite immune reconstitution in the peripheral blood. Though the mechanism remains obscure, the data suggest that there is either viral or immune-mediated accelerated T cell destruction or, possibly, alterations in T cell homing to the GI tract. Although clinically silent over the short term, the long-term consequences of the persistence of this lesion may emerge as the HIV-1–infected population survives longer owing to the benefits of HAART.

## Abstract 2

Despite early initiation of anti-HIV therapy, loss of T cells in the gastrointestinal mucosa persisted for years in most members of a clinical cohort identified early after HIV-1 infection.

## Editors' Summary

**Background.:**

AIDS causes disease by inactivating the body's immune response against infection. The AIDS virus (HIV) is most active against the white blood cells called T lymphocytes, particularly the CD4 T lymphocytes, which recognize infection and activate other cells of the immune system to fight it. In what was formerly believed to be a gradual process, HIV infection is now known to deplete a subset of the body's CD4 lymphocytes, called memory cells, quite rapidly—over only a few days—within a few weeks after a person becomes infected with the AIDS virus. This was not known until recently because researchers were counting CD4 cells only in blood, while a majority of the memory lymphocytes are located in and around the digestive system. It is these intestinal memory lymphocytes that are rapidly wiped out, while those in the blood fall much more gradually, usually over several years. Few studies of mucosal lymphocytes have been done in humans because such studies require biopsies of the intestinal lining (mucosa).

**Why Was This Study Done?:**

Although CD4 cells in the blood can return and remain at normal levels when HIV infection is treated with antiviral drugs, it has been unclear as to whether the mucosal CD4 cells return as well. People who begin treatment as soon as possible after becoming infected with HIV might seem to have the best chance of regaining their mucosal immunity, compared to those who wait until the CD4 cells in their blood have fallen, which is a generally accepted reason to start medication for HIV. Therefore, the researchers wanted to see whether people who start treatment early after becoming infected with HIV might experience restoration of their mucosal immunity over time and, if so, what kinds of lymphocytes would return.

**What Did the Researchers Do and Find?:**

The researchers studied people who started treatment for HIV within a few weeks to months of becoming infected and who then remained on treatment. Some volunteers underwent biopsies of the intestinal mucosa before starting treatment and then at various points from 1 y until as long as 3 y after infection. Others volunteered for biopsy only one time, anywhere from less than 1 y to 7 y following treatment. The biopsy specimens were examined under the microscope and with a technique called flow cytometry using specific staining methods to assess their structure and functional characteristics. Results were compared to biopsies from a group of HIV-uninfected volunteers.

The researchers found that the percentage of CD4 lymphocytes dropped much lower in the intestinal mucosa than in blood during early infection and then, unlike in blood, remained low even after several years of treatment for HIV. In the microscope images, they found that mucosal CD4 cells were lost mostly from regions of active battle against invading germs, rather than from “training sites” for new CD4 cells. Over time, only 30% of the volunteers showed return of CD4 cells to normal levels in these active sites.

Unlike T lymphocytes in the blood, which tend to return to a resting state after HIV is treated, the T lymphocytes in the intestinal mucosa tended to persist in an activated state despite HIV treatment, even though only a tiny fraction of these cells were found to be infected with HIV. A high level of activation of mucosal lymphocytes soon after infection was found to predict poor restoration of mucosal CD4 cells over time.

**What Do These Findings Mean?:**

These experiments confirm that studying easily obtained blood lymphocytes provides only a limited view of how HIV affects the immune system as a whole. The finding that immune cells of the intestinal mucosa remain depleted and over-activated for years despite antiretroviral treatment raises the concern that over time this will result in clinical problems. Fortunately, this does not appear to be the case in most people currently being treated for HIV, some for as long as 10 y, but the results of this study suggest that we should remain vigilant for gastrointestinal problems resulting from impaired immunity over time. The finding that mucosal lymphocytes do appear to return to normal levels in a minority of volunteers is of interest, and suggests that early interventions to reduce activation of intestinal T cells (such as antimicrobial or immunomodulatory treatment) might be worth investigating in those recently infected with HIV. Finally, these results suggest that a vaccine to prevent HIV may need to stimulate immune responses that can act very quickly following infection, before the bulk of lymphocytes capable of countering the infection are lost, perhaps irreversibly.

**Additional Information.:**

Please access these Web sites via the online version of this summary at http://dx.doi.org/10.1371/journal.pmed.0030484.

- UCSF HIV InSite includes resources on HIV immunology and vaccine development
- AIDS fact sheets and brochures from the US National Institute of Allergy and Infectious Diseases
- Medline Plus article on acute HIV infection from the US National Library of Medicine

## Introduction

Over the past 25 y, more than 25 million individuals have succumbed to the complications of HIV-1 infection [1]. The development and evolution of highly active antiretroviral therapy (HAART) has resulted in the ability to achieve durable control of viral replication and preservation of immune function in the majority of treated patients with relatively simple and minimally toxic treatment regimens [2–7]. Recent findings suggest that immune parameters measured in the peripheral blood of patients infected with HIV-1 may not be representative of the immune system as a whole, particularly when considering the status of the less well characterized, but far more sizeable mucosal immune system [8–11].

During acute and early HIV-1 infection (AEI), there is selective depletion of CD4^+^ T cells in the gastrointestinal (GI) lamina propria (LP) compared with levels measured in the peripheral blood [10–12]. Our studies have suggested that up to 60% of LP CD4^+^ T lymphocytes are lost as early as 2–3 wk into the course of infection. This is likely due to the presence of densely clustered CCR5^+^ memory CD4^+^ T cells [13–15] at mucosal sites that serve as preferred targets for HIV-1 infection.

These findings have reignited interest in the impact of HAART on CD4^+^ T cell reconstitution in the GI tract compared with the peripheral blood. Guadalupe et al. have reported that of two patients treated during primary HIV-1 infection, near-complete mucosal reconstitution occurred in one patient, whereas the second patient showed incomplete restoration despite 5 y of antiretroviral therapy [12]. The same group concluded from the study of simian immunodeficiency virus (SIV)–infected macaques that CD4^+^ T cell reconstitution could be achieved to near-complete levels in the GI tract if antiretroviral therapy was initiated during primary SIV infection but not if therapy was started later in infection [16]. Our initial description of eight patients studied cross sectionally 6 mo to 5 y after the initiation of therapy during acute and early infection suggested that despite therapy, CD4^+^ T cell depletion persisted in the GI mucosa [11]. Thus, there are somewhat conflicting and limited data on the reconstitution of the GI immune system with potent antiretroviral therapy, particularly when treatment is initiated during AEI. Given that the GI tract harbors the largest collection of immune cells in the body, it is critical to assess this compartment for immune reconstitution with antiretroviral therapy.

To address this issue conclusively, we undertook the present study. Our aims were: (1) to determine the effect of uninterrupted antiretroviral therapy initiated during AEI on the reconstitution of GI mucosal T-lymphocyte population; (2) to examine the phenotype of mucosal lymphocytes prior to and during long-term antiretroviral therapy; and (3) to examine clinical, immunological, and virological factors involved in the reconstitution of the GI immune system during antiretroviral therapy. We prospectively followed 18 individuals identified and treated during AEI, and performed recto-sigmoid colonic biopsies on these individuals prior to treatment and serially up to 32 mo following the initiation of treatment. In addition, we extended our cross-sectional colonic biopsy studies to include a total of 22 individuals who were also treated during acute and early infection. We therefore examined a relatively large cohort of patients, all treated during AEI, to determine whether CD4^+^ T cell numbers reconstitute to the same extent in the GI tract as observed in the peripheral blood. An additional 14 individuals with AEI provided biopsy specimens only prior to treatment.

## Methods

### Patients and Sample Acquisition

Peripheral blood and recto-sigmoid colonic mucosal tissue were collected from HIV-1–infected patients and HIV-1–uninfected control participants. Informed consent was obtained from all patients and the study was approved by the Institutional Review Boards of the Rockefeller University, Bellevue Hospital Center (New York, New York, United States), and Manhattan Veteran's Administration Hospital Center (New York, New York, United States). All clinical investigation was conducted according to the principles expressed in the Helsinki Declaration.

Endoscopic biopsies were obtained from macroscopically normal colonic mucosa and were processed as described previously [11]. Briefly, the biopsies were taken using large-cup endoscopic-biopsy forceps (Microvasive Radial Jaw, Boston Scientific, Boston, Massachusetts, United States) (outside diameter 3.3 mm) and (1) placed immediately in tissue-culture medium (RPMI 1640, Mediatech, Herndon, Virginia, United States); (2) placed into 2-ml pre-labeled cryovials (Nalgene, Rochester, New York, United States) and frozen in liquid nitrogen; or (3) placed in formalin to preserve tissue architecture. Formalin-fixed tissues were washed with phosphate-buffered saline (PBS), transferred to 100% alcohol and processed for immunohistochemistry and in-situ hybridization. Phlebotomy was undertaken immediately prior to endoscopy.

Immediately after acquisition, mucosal mononuclear cells (MMCs) were enzymatically isolated from mucosal biopsies using a 30-min incubation in collagenase type II (Clostridiopeptidase A, Sigma-Aldrich, St. Louis, Missouri, United States) followed by mechanical separation through a blunt-ended 16-gauge needle. The digested cell suspension was strained through a 70-μm disposable plastic strainer. Immediately after isolation, cells were washed with PBS and resuspended in PBS containing antibodies for flow cytometry. Peripheral blood mononuclear cells (PBMCs) were prepared by centrifugation on a Ficoll-Hypaque density gradient (Mediatech). PBMCs were stained for flow cytometry immediately after isolation.

### Flow Cytometry

Cell surface expression of lymphocyte antigens was identified by monoclonal antibody staining of freshly isolated MMCs and PBMCs, followed by flow cytometry using a FACSCalibur (Becton-Dickinson, Palo Alto, California, United States) with analysis using CellQuest software (Becton-Dickinson). Monoclonal antibodies used in this study included: anti-human CD3-fluorescein isothiocyanate (FITC) (clone UCHT1) (Becton-Dickinson), anti-human CD3-phycoerythrin (PE) (clone SK-7) (Becton-Dickinson), anti-human CD3-peridinin chlorophyll-α protein (PerCP) (clone SK-7) (Becton-Dickinson), anti-human CD4-allophycocyanin (clone RPA T4) (PharMingen, San Diego, California, United States), anti-human CD8 PE (clone RPA T8) (PharMingen), anti-human CXCR4-PE (clone 12G5) (PharMingen), anti-human CCR5-FITC (clone 2D7/CCR5) (PharMingen), anti-human HLA-DR PerCP (clone L243, BD Biosciences Pharmingen, San Diego, California, United States), anti-human CD45RO PE (clone UCHL1, BD Biosciences Pharmingen), anti-human Ki67 FITC (clone B56, BD Biosciences Pharmingen), anti-human CCR7 PE (clone 3D12, BD Biosciences Pharmingen), anti-human CD62L allophycocyanin (clone Dreg56, BD Biosciences Pharmingen), and the appropriate isotype controls. During flow cytometry, lymphocytes, initially identified by their forward- and side-scatter characteristics, were subject to phenotypic analysis. Dead cells were excluded from analysis using 7-aminoactinomycin D [17] (Calbiochem, San Diego, California, United States).

To determine the percentages of CD4^+^ and CD8^+^ cells in the T cell population, gated lymphocytes were initially examined for the expression of CD3. The CD3^+^ lymphocytes were then analyzed for expression of CD4 and CD8 receptors. To evaluate the expression of chemokine co-receptors, gated lymphocytes were initially examined for the expression of CD4 receptors. The CD4^+^ lymphocytes were further examined for the expression of chemokine co-receptors CCR5 and CXCR4. To examine for activated memory cells, gated CD4^+^ and CD8^+^ lymphocytes were examined for the expression of CD45RO and HLA-DR. Central and effector memory cells were evaluated by the expression of CD62L and CCR7 on gated CD4^+^ and CD8^+^ lymphocytes.

### Light Microscopy and Immunohistochemistry

All immunohistochemistry and in-situ hybridization sections were examined independently by two board-certified pathologists with significant experience in the field.

For light-microscopic evaluation, tissues were fixed in 4% neutral-buffered formalin and embedded in paraffin. Sections (of 5 μm thickness) were cut and stained with hematoxylin-and-eosin and Giemsa stains. Immunohistochemistry was also performed on paraffin-embedded sections after high-temperature antigen retrieval as described previously [18]. Briefly, the sections were de-waxed and antigen retrieval was performed by pressure cooking the sections for 3 min in 50 mM Tris and 2 mM EDTA (pH 9)—for CD4, or 3 min in 0.01 M buffered sodium citrate solution (pH 6)—for CD8. The sections were cooled to room temperature and rinsed in Tris-buffered saline (pH 7.4). The sections were incubated with 1:25 dilution of antibody to CD4 (NCL-CD4-IF6, Novocastra Laboratories, Newcastle-upon-Tyne, United Kingdom) or to 1:100 dilution of antibody to CD8 (C8/144B, DakoCytomaton, Glostrup, Denmark) for 60 min, followed by incubation with a 1:20 dilution of rabbit anti-mouse secondary antibody (DakoCytomaton code 259) for 20 min. The tertiary antibody (APAAP-Complex Monoclonal Mouse, DakoCytomaton) was applied in 1:50 dilution. The incubations were carried out at room temperature and were followed by rinsing in Tris-buffered saline (pH 7.4) for 5 min each. The alkaline phosphatase was revealed by New Fuchsin as the chromogen.

CD4^+^ or CD8^+^ cells in the LP (effector site) and the organized lymphoid tissue (OLT) (inductive site) were quantified separately. Using a 40× objective, a standard area was set (unit area), and a photomicrograph was taken with a Zeiss AxioImager M1 microscope equipped with AxioCam MRc5 digital camera (Zeiss, Jena, Germany). Fifteen nonoverlapping unit areas were selected for the LP, and between two and five unit areas were selected for the OLT, depending upon the size of the T-dependent zone. Using AxioVision (Release 4.5) software (Zeiss), positive cells showing lymphocyte morphology were counted. Because macrophages and dendritic cells also express CD4 in high intensity, manual counting was chosen instead of automatic measurement. Owing to technical reasons (high background signal, lack of adequate material, inadequate staining, etc.), data were available only on 30 out of the 40 patients.

### Immunohistochemical Double Labeling for Detection of Proliferating T Cells

After heat-mediated antigen retrieval by pressure cooking (3 min in 50 mM Tris and 2 mM EDTA [pH9]), the sections were incubated with anti-CD4 or CD8 antibodies overnight as described above. Immunodetection was performed either with the StreptABComplex/HRP (Code K0391, DakoCytomaton) using 3-amino-9-ethylcarbazole (Sigma-Aldrich) as the substrate or with APAAP (DakoCytomaton) and Fast Blue as chromogen. The sections were then heat-treated again for 5 min with 0.01 M buffered sodium citrate solution (pH 6.0). This was followed by an overnight incubation with MIB-1 (anti-Ki67 antibody, DakoCytomaton). For the second antibody, either the APAAP (DakoCytomaton) or the StreptABComplex/HRP (DakoCytomaton) visualization system was applied.

### In Situ Hybridization

The in situ hybridization was performed on paraffin sections as described previously [18]. A ^35^S-labeled, single-stranded anti-sense RNA probe of HIV-1 (Lofstrand Laboratories, Gaithersburg, Maryland, United States), composed of fragments of 1.4–2.7 kb, which collectively represent approximately 90% of the HIV-1 genome [19], was used. Multiple sections (4–28) were examined in the treated cases to detect the presence of HIV-1 RNA–expressing cells.

### Semiquantitative Analysis of the HIV-1 RNA–Positive Cells

The autoradiographs were examined with a microscope equipped with epiluminescent illumination (Axiophot, Zeiss), a 3CD camera, and a PC-based image-analysis system (KS 4000, Kontron, Esching, Germany) as described previously [11]. Briefly, cells were considered positive for viral gene expression if the grain count was more than six times that of the background. The positive cells were counted. The area occupied by lymphoid follicles was measured, and the frequency of RNA-producing cells per mm^2^ of tissue was calculated. To evaluate the number and distribution of T cell subsets, transmission light was used. Using a 40× objective, a standard area was set by the image analyzer. The number of positive cells within this unit area was determined by manual counting. For the LP, a total of 10–15 consecutive nonoverlapping fields were analyzed for each staining. For the OLT, between two and five representative areas were chosen. The individual values obtained with each T cell marker were then pooled, and the mean numbers of positive cells per unit area of LP or OLT were determined separately.

### Statistical Methodology

Values are expressed as mean ± standard deviation. Statistical comparisons were made between PBMCs and MMCs from individuals using a paired *t*-test. Statistical comparisons were made between HIV-1–infected patients and control participants using a two-sample, unequal variance *t*-test. All reported *p*-values were two-sided at the 0.05 significance level using SPSS 11.0 for Windows software (SPSS, Chicago, Illinois, United States).

## Results

### Baseline Characteristics of Study Patients

A total of 54 patients with AEI and 18 HIV-1–uninfected control participants were studied. Of the HIV-1–infected patients, biopsies were performed on 32 individuals during AEI prior to initiation of antiretroviral therapy. Of these 32 patients, 18 were followed longitudinally post–antiretroviral therapy initiation with recto-sigmoid biopsies at 1 y (*n* = 5), 2 y (*n* = 9), and 3 y (*n* = 4). An additional 22 patients were studied cross sectionally after initiation of HAART for up to 1 y (*n* = 7), 2 y (*n* = 7), and 3–7 y (*n* = 8) (Table 1). On presentation, patients were all viremic and were staged as per the National Institutes of Health (NIH)–sponsored Acute HIV Infection and Early Disease Research Program: enzyme immunoassay (EIA)–negative (stage Ia), Western blot indeterminate (stage Ib), a nonreactive detuned EIA [20] with an optical density (OD) value of less than 0.5 or a documented negative EIA within 3 mo of presentation (stage II), and a nonreactive detuned EIA (OD 0.51–1.0) or a documented negative serology within 6 mo of presentation (Stage III). During AEI, all patients had initiated therapy that comprised combinations of nucleoside (nucleotide) reverse transcriptase inhibitors with either a protease inhibitor and/or a non-nucleoside reverse transcriptase inhibitor. Patients were highly adherent to treatment and maintained undetectable plasma viral loads during treatment. There were no significant differences between the longitudinal and cross-sectional groups with respect to CD4^+^ T cell count on presentation, log_10_ HIV-1 viral load on presentation, or the estimated duration of infection on presentation. All were male patients who had contracted HIV-1 sexually during same-sex contact.

**Table 1:**
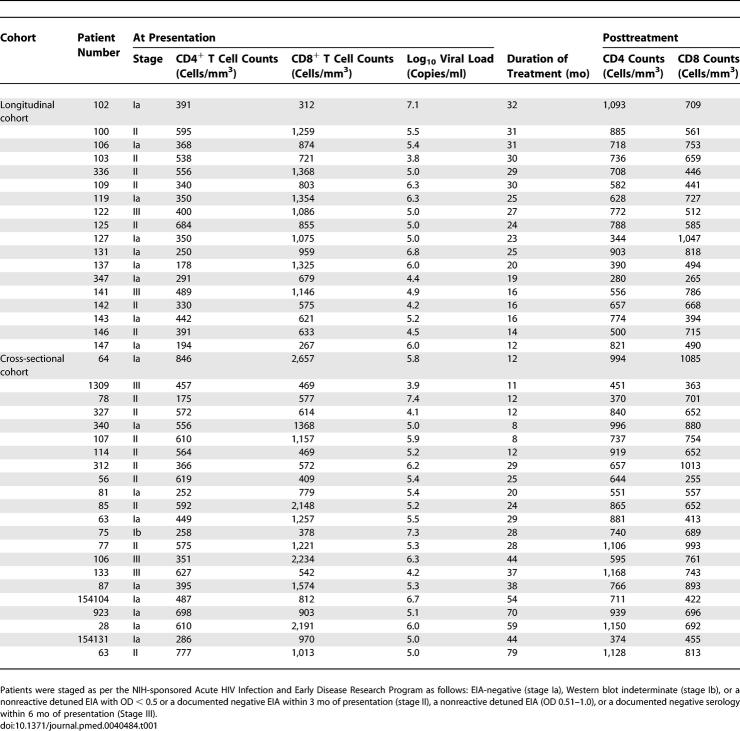
Patient Characteristics

The 18 HIV-1–uninfected control participants were recruited from a population undergoing screening colonoscopy at the time of study recruitment. This group comprised ten men and eight women. None of the HIV-1–infected patients or HIV-1–uninfected control participants were found to have macroscopic evidence of GI mucosal disease, nor were any concomitant pathological processes found on histological examination. All enrolled patients and control participants signed an informed-consent form that was approved by the institutional review boards of the Rockefeller University, Bellevue Hospital Center, and Manhattan Veteran's Administration Hospital Center.

### Reconstitution of CD4^+^ T Cells Is Incomplete despite HAART

**Figure 1:**
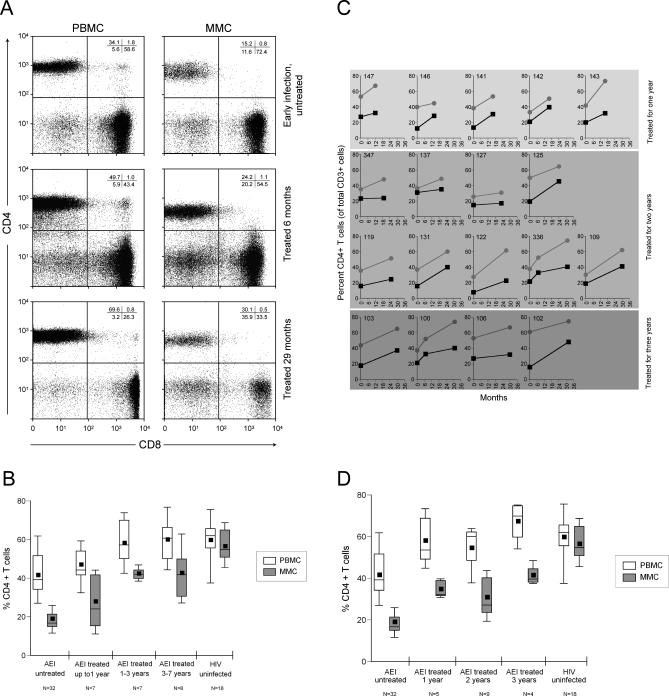
Persistent Depletion of CD4^+^ T cells in the GI Tract despite Normalization in the Peripheral Blood PBMCs and MMCs from 54 patients with AEI and 18 HIV-1–uninfected control participants were analyzed by flow cytometry. CD3^+^ gated lymphocytes were analyzed for the expression of CD4 and CD8. (A) Representative flow plots from patient 336 are depicted. CD8^+^ T cells are shown on the *x*-axis and CD4^+^ T cells on the *y*-axis. (B) A box plot depicting the comparison between CD4^+^ T cells derived from the blood and GI tract of 22 patients examined cross sectionally. The percentages of CD4^+^ T cells in the blood (white) and GI tract (grey) are compared between HIV-uninfected, AEI-untreated, and AEI groups treated for up to 1 y, 1–3 y, and 3–7 y, respectively. In these plots, the boxes extend from the first to the third quartiles, enclosing the middle 50% of the data. The middle line within each box indicates the median of the data, whereas the vertical line extends from the 10th to the 90th percentile. Means of the data are represented by filled-in squares. (C) Comparisons between the blood and GI tract of 18 patients followed longitudinally after initiation of antiretroviral therapy. The percentages of CD4^+^ T cells in the blood (grey) and GI tract (black) at baseline (AEI-untreated) and following treatment are depicted per study patient. (D) Cumulative data from the 18 patients followed longitudinally where the percentages of CD4^+^ T cells in the blood (white) and GI tract (grey) are compared after 1 y, 2 y, and 3 y of HAART.

In order to study the effect of antiretroviral therapy on the reconstitution of CD4^+^ T cells, we utilized flow cytometry to determine the percentage of CD4^+^ T cells in the GI tract and peripheral blood (Figure 1A). Twenty-two HIV-1–infected, treated patients were examined cross sectionally (where no pre-ART biopsy was available) and were compared with HIV-infected, untreated patients (AEI patients, *n* = 32) and uninfected control participants (*n* = 18). In the uninfected control participants, the mean PBMC CD4^+^ T cell percentage was 59.6% ± 14.3% and the mean MMC CD4^+^ T cell percentage was 56.4% ± 8.8%. In AEI patients, the mean PBMC CD4^+^ T cell percentage was 41.5% ± 12.9% and the mean MMC CD4^+^ T cell percentage was 19.3% ± 8.8%. In patients treated for up to 1 y (*n* = 7), the mean PBMC CD4^+^ T cell percentage was 46.9% ± 10.1% and the mean MMC CD4^+^ T cell percentage was 27.8% ± 14.5%. In patients treated for 1–3 y (*n* = 7), the mean PBMC CD4^+^ T cell percentage was 58.1% ± 12.3% and the mean MMC CD4^+^ T cell percentage was 42.3% ± 3.1%. In patients treated for 3–7 y (*n* = 8), the mean PBMC CD4^+^ T cell percentage was 59.9% ± 12.0% and the mean MMC CD4^+^ T cell percentage was 42.5% ± 13.6% (Figure 1B). Thus, based on flow cytometry, in the cross-sectional group, the percentage of CD4^+^ T cells in the GI tract was significantly lower than the percentage of peripheral blood CD4^+^ T cells during AEI (*p* < 0.001), and remained low after treatment for up to 1 y (*p* < 0.001), 1–3 y (*p* = 0.003), and 3–7 y (*p* = 0.02).

Next, we sought to follow patients longitudinally to assess the effect of antiretroviral therapy on the GI tract and peripheral blood in each study participant. Of the 32 patients that were examined during AEI, we followed 18 patients with serial biopsies over a span of 3 y. Since patient recruitment for repeated intestinal biopsies is challenging, enrolment in the longitudinal group was limited to five out of 18 at year 1, nine out of 18 at year 2, and four out of 18 at year 3 of antiretroviral treatment. In each individual studied, the percentage of CD4^+^ T cells remained significantly lower in the GI tract compared with the peripheral blood (Figure 1C). When assessed as a group, the difference between PBMC and MMC CD4^+^ T cell percentage was highly significant after 1 y (57.9% ± 11.9% CD4^+^ PBMCs versus 34.6% ± 4.2% CD4^+^ MMCs; *p* < 0.001), 2 y (54.4% ± 10.9% CD4^+^ PBMCs versus 30.8% ± 9.8% CD4^+^ MMCs; *p* < 0.001), and 3 y (67.3% ± 9.8% CD4^+^ PBMCs versus 41.3% ± 4.9% CD4^+^ MMCs; *p* = 0.02) of suppressive antiviral therapy (Figure 1D). Thus, based on flow cytometry, the percentage of CD4^+^ T cells remained significantly lower in the GI tract compared with the peripheral blood despite protracted antiretroviral therapy.

### Immune-Inductive Sites Show Reconstitution with Therapy, whereas Immune-Effector Sites Show Persistent CD4^+^ T Cell Depletion

**Figure 2:**
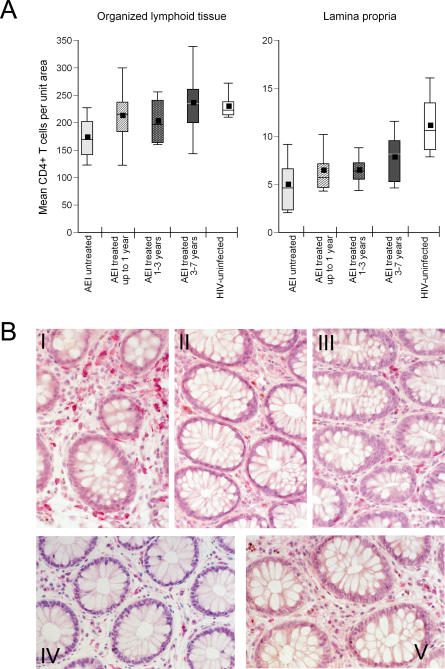
Effector Sites (LP) of the GI Tract Showing Persistent CD4^+^ T Cell Depletion during HAART Immunohistochemical characterization of immune-inductive and effector sites in rectal biopsies. Using a PC-based image-analysis system (KS 4000, Kontron) a standard area was set by the image analyzer. For the LP, a total of between ten and 15 consecutive nonoverlapping fields were analyzed for each staining. For the OLT, between two and five representative areas were chosen. (A) CD4^+^ T cells per unit area were determined in OLT (left panel) and LP (right panel). Mean CD4^+^ T cell numbers were compared between HIV-uninfected (white boxes), AEI (light grey boxes), and patients treated for up to 1 y (hatched boxes), 1–3 y (speckled boxes) and 3–7 y (dark grey boxes). In these plots, the boxes extend from the first to the third quartiles, enclosing the middle 50% of the data. The middle line within each box indicates the median of the data, whereas the vertical line extends from the 10th to the 90th percentile. Means of the data are represented by filled-in squares. (B) A biopsy section (viewed at 40× magnification) from an HIV-uninfected control participant, showing CD4^+^ T cells (stained red) within the GI LP (panel I). In contrast, a pronounced reduction in LP CD4^+^ T cells is noted in a patient with AEI (patient no. 131) in panel II which does not correct despite antiretroviral therapy for 2 y in the same patient (panel III). Another representative study patient (no. 142) is presented, where LP CD4^+^ T cell depletion during AEI (panel IV) does not correct after antiretroviral therapy for 1 y (panel V).

To corroborate flow cytometry–derived data and to assess for the absolute numbers of mucosal CD4^+^ T cells, immunohistochemistry was performed on paraffin-embedded biopsy sections. Since CD4^+^ T cell depletion occurs preferentially in the LP [11], we examined the immune-inductive sites (represented by OLT) and effector sites (LP) for the effect of antiretroviral therapy separately (Figure 2). All patients (studied cross sectionally and longitudinally, data available on 30 out of 40 patients) are described in Figure 2. In HIV-uninfected control participants, the mean CD4^+^ T cell count was 229.6 ± 24.6 cells/unit area in OLT and 11.0 ± 3.3 cells/unit area in LP. By comparison, mean CD4^+^ T cell counts in the OLT were 212.5 ± 63.8, 202.5 ± 46.2, and 235.9 ± 69.6 cells/unit area in the patients treated for up to 1 y, 1–3 y, and 3–7 y, respectively. However, in the LP, mean CD4^+^ T cell counts statistically were highly significantly depleted in all groups—6.4 ± 2.3 (*p* < 0.001), 6.5 ± 1.6 (*p* < 0.001), and 7.8 ± 2.6 cells/unit area (*p* = 0.004)—in the three treatment groups, respectively. On examination of individual patients, nine showed “normalization” (defined as mean CD4^+^ T cells per unit area in the LP of 18 HIV-uninfected control participants—11.0 ± 3.3 cells/unit area). The mean LP CD4^+^ T cell count in these nine patients was 9.5 ± 1.4 cells (*p* = 0.13 compared with HIV-uninfected), the mean LP CD8^+^ T cell count was 8.4 ± 2.7 cells (*p* = 0.3), and the mean CD4:CD8 ratio in the LP was 1.2 ± 0.6 (*p* = 0.07).

In the other 21 patients, persistent depletion of CD4^+^ T cells was noted in the LP, with a mean CD4^+^ T cell count of 5.5 ± 1.1 cells (*p* < 0.001). The mean CD8 T cell count in these 21 “non-reconstitutors” was 8.0 ± 1.7 cells (*p* = 0.3) and the mean CD4:CD8 ratio was 0.7 ± 0.1 (*p* < 0.001). Thus, significant depletion of the GI CD4^+^ T cell count persisted in 21 out of 30 (70%) of patients despite uninterrupted, apparently suppressive, combination antiretroviral therapy. As a corollary, in nine out of 30 (30%) of patients, the absolute CD4^+^ T cell count per unit area reached HIV-uninfected levels with treatment.

### CCR5^+^ and CCR5^+^/CXCR4^+^ CD4^+^ T Cells in the GI Tract Remain Depleted Despite Therapy

**Figure 3:**
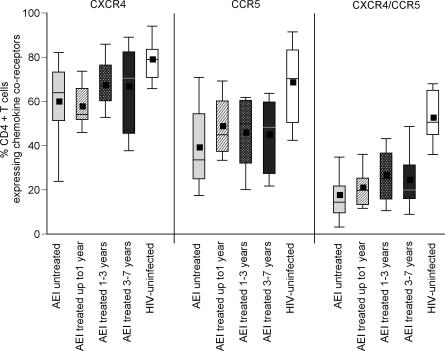
CD4^+^ T Cells Expressing CCR5 and CCR5/CXCR4 Are Preferentially Depleted from the GI Tract during HAART CD3^+^/CD4^+^ gated MMCs were analyzed for the expression of chemokine receptors CCR5, CXCR4, and CCR5/CXCR4 in HIV-uninfected (white boxes), AEI (light grey boxes), and patients treated for up to 1 y (hatched boxes), 1–3 y (speckled boxes), and 3–7 y (dark grey boxes). In these plots, the boxes extend from the first to the third quartiles, enclosing the middle 50% of the data. The middle line within each box indicates the median of the data, whereas the vertical line extends from the 10th to the 90th percentile. Means of the data are represented by filled-in squares.

Since a majority of the viruses during AEI are CCR5-tropic [21], and a significant proportion of cells in the GI tract express CCR5 [13,14], depletion of CCR5-expressing cells is observed preferentially in the GI tract during AEI [11,12]. We wanted to assess the impact of antiretroviral therapy on the reconstitution of CD4^+^ T cells expressing chemokine receptors. Using four-color flow cytometry, we quantified the percentage of CD3^+^/CD4^+^ MMCs that expressed CCR5 or CXCR4, or co-expressed CCR5 and CXCR4 (Figure 3) in all patients (followed longitudinally and cross sectionally on HAART, *n* = 40). Comparisons were made between HIV-uninfected control participants, AEI, AEI treated for up to 1 y, AEI treated for 1–3 y, and AEI treated for 3–7 y. In the HIV-uninfected patients, 78.8% ± 10.3% CD4^+^ T cells expressed CXCR4, 68.4% ± 18.9% expressed CCR5, and 52.4% ± 12.4% co-expressed CXCR4/CCR5. As reported previously [11], depletion in AEI untreated patients was noted in CD4^+^ MMCs expressing CXCR4 (59.7% ± 20.0%, *p* = 0.04), more so in CD4^+^CCR5^+^ T cells (39.0% ± 20.0%, *p* = 0.002), and the most significantly in CD4^+^ MMCs dually expressing both CCR5 and CXCR4 chemokine receptors (17.4% ± 14.2%, *p* < 0.001).

Despite antiretroviral therapy, significant depletion persisted in the CD4^+^CCR5^+^ and CD4^+^ dual-positive MMCs, when compared with HIV-uninfected patients. Specifically, CD4^+^CCR5^+^ MMCs in patients treated for up to 1 y (48.6% ± 14.7%, *p* = 0.01), 1–3 y (45.7% ± 19.4%, *p* = 0.01), and 3–7 y (44.7% ± 17.6%, *p* = 0.01) remained depleted. Similarly, though more prominently, levels of CD4^+^CCR5^+^/CXCR4^+^ MMCs were lower in patients treated for up to 1 y (20.8% ± 8.4%, *p* < 0.001), 1–3 y (26.6% ± 15.8%, *p* = 0.001), and 3–7 y (24.4% ± 13.8%, *p* < 0.001), respectively.

### Activated Memory CD4^+^ and CD8^+^ T Cells Remain Elevated in the GI Tract during HAART

HIV-1 infection results in a significant increase in cellular activation, which has been shown to be of prognostic value in predicting the rate of CD4^+^ T cell decline without therapy [22–24]. We wanted to understand the impact of cellular activation at baseline and following treatment on the reconstitution of CD4^+^ and CD8^+^ T cells in the GI tract and peripheral blood. Using four-color flow cytometry, we assessed CD3^+^CD4^+^ and CD3^+^CD8^+^ lymphocytes for the expression of CD45RO, a marker of immunological memory, and HLA-DR, a marker of immunological activation.

It is well established that a majority of mucosal cells (>90%) have a memory (CD45RO^+^) phenotype [15]. Consistent with this finding, we observed that 92.1% ± 10% of GI CD4^+^ T cells were CD45RO^+^ in HIV-uninfected control participants. In patients with untreated AEI, the percentage of GI CD4^+^CD45RO^+^ T cells showed only a modest reduction to 86.3% ± 12%, *p* = 0.17 despite a well-documented, profound depletion of the total number of GI CD4^+^ T cells. Thus, the relative percentage of memory cells in the GI tract remained stable despite an overall reduction in the total number of CD4^+^ T cells. To examine the levels of cellular activation, we examined memory (CD45RO^+^) lymphocytes for the expression of HLA-DR.

**Figure 4:**
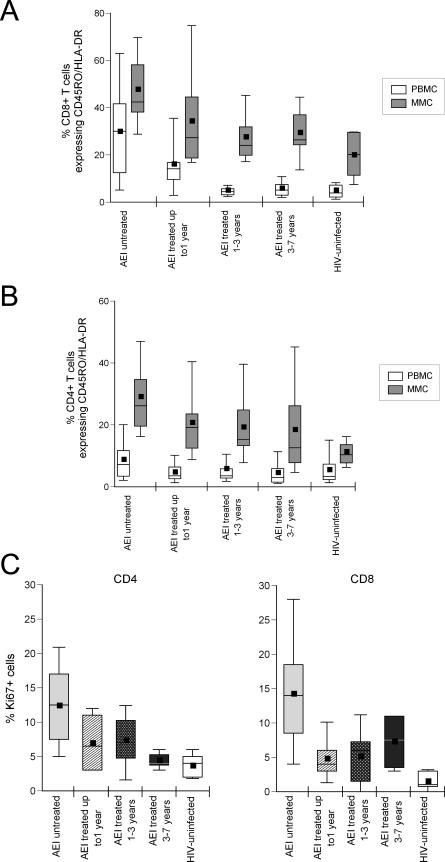
The Level of Activated and Proliferating CD4^+^ and CD8^+^ T Cells Remains Elevated in the GI Tract during HAART Using four-color flow cytometry, co-expression of CD45RO and HLA-DR was examined on CD3^+^CD8^+^ (A) and CD3^+^CD4^+^ (B) gated lymphocytes in HIV-uninfected control participants, AEI, and patients treated for up to 1 y, 1–3 y, and 3–7 y. PBMCs are depicted in white and MMCs in grey boxes. (C) Using immunohistochemistry, the percentage of GI cells expressing Ki67 was determined on CD4^+^ T cells (left panel) and CD8^+^ T cells (right panel). Levels in HIV-uninfected control participants (white boxes) were compared with AEI (light grey boxes) and patients treated for up to 1 y (hatched boxes), 1–3 y (speckled boxes), and 3–7 y (dark grey boxes). In these plots, the boxes extend from the first to the third quartiles, enclosing the middle 50% of the data. The middle line within each box indicates the median of the data, whereas the vertical line extends from the 10th to the 90th percentile. Means of the data are represented by filled-in squares.

In the HIV-uninfected control participants, CD8^+^CD45RO^+^HLA-DR^+^ PBMCs represented 4.8% ± 3.9% of the population and CD8^+^CD45RO^+^HLA-DR^+^ MMCs represented 19.8% ± 9.8% of the population. In comparison, during AEI, a significant increase in CD8^+^CD45RO^+^HLA-DR^+^ PBMCs (29.6% ± 19.5%, *p* < 0.001) and CD8^+^CD45RO^+^HLA-DR^+^ MMCs (47.57% ± 16.8%, *p* < 0.001) was noted. After 1 y of treatment, the percentage of CD8^+^CD45RO^+^HLA-DR^+^ PBMCs remained elevated (15.9% ± 12.1%, *p* = 0.006) but approximated HIV-uninfected levels in the 1–3 y treated (4.9% ± 2.9%, *p* = 0.9) and 3–7 y treated groups (5.7% ± 3.6%, *p* = 0.5). In contrast, in the GI tract, the percentage of CD8^+^CD45RO^+^HLA-DR^+^ MMCs remained elevated despite HAART in all groups—1 y treated (34.1% ± 20.2%, *p* = 0.02), 1–3 y treated (27.4% ± 10.7%, *p* = 0.04), and 3–7 y treated (29.3% ± 11.6%, *p* = 0.04) (Figure 4A).

Similar, though less pronounced, results were noted in the activated memory CD4^+^ T cell subsets. CD3^+^CD4^+^ PBMCs co-expressing CD45RO/HLA-DR were 5.4% ± 5.0% of the CD4 population in the HIV-uninfected control participants. During AEI, there was a significant increase in this population (8.7% ± 6.5%, *p* = 0.04). Post-HAART, activated memory CD4^+^ PBMCs approximated HIV-uninfected levels in all groups (1 y treated group: 4.6% ± 3.2%, *p* = 0.6; 1–3 y treated group: 5.7% ± 6.5%, *p* = 0.8; and 3–7 y treated group: 4.5% ± 4.4%, *p* = 0.6). In the GI tract, however, activated memory CD4^+^ T cells persisted despite therapy. When compared with HIV-uninfected CD4^+^CD45RO^+^HLA-DR^+^ MMCs (11.3% ± 5.0%), an increase in activated memory CD4^+^ T cells that developed during AEI (29.03% ± 12.1%, *p* < 0.001) persisted despite potent antiretroviral therapy in the 1 y treated group (20.6% ± 10.6%, *p* = 0.007) and 1–3 y treated group (19.2% ± 10.6%, *p* = 0.01), but not in the 3–7 y treated group (18.3% ± 15.2%, *p* = 0.1) (Figure 4B).

To study a more specific marker of cellular dynamics, we examined the expression of Ki67 on GI mucosal CD4^+^ T cells and CD8^+^ T cells by immunohistochemistry (Figure 4C). Compared with uninfected control participants (3.6% ± 1.6% Ki67^+^CD4^+^ T cells and 1.5% ± 1.3% Ki67^+^CD8^+^ T cells), during untreated AEI, a significant increase was noted in the percentage of Ki67^+^CD4^+^ T cells (12.4% ± 6.1%, *p* < 0.001) and CD8^+^ T cells (14.2% ± 4.7%, *p* < 0.001). This increase persisted in the group treated for 1 y (6.9% ± 3.8%, Ki67^+^CD4^+^ T cells, *p* < 0.02 and 4.7% ± 3.2% Ki67^+^CD8^+^ T cells, *p* < 0.02) and 1–3 y (7.3% ± 3.9%, Ki67^+^CD4^+^ T cells, *p* < 0.007 and 5.1% ± 4.0% Ki67^+^CD8^+^ T cells, *p* < 0.007). In the 3–7 y treated group, the increase in the percentage of Ki67^+^ cells persisted (4.4% ± 1.4% Ki67^+^CD4^+^ T cells, *p* = 0.27 and 7.25% ± 4.3% Ki67^+^CD8^+^ T cells, *p* = 0.07), although it was not statistically significant.

Thus, while CD4^+^ and CD8^+^ lymphocyte activation reverses with treatment in the peripheral blood, it remains elevated in the GI tract. In addition, there is a persistent increase in proliferating CD4^+^ and CD8^+^ T cells in the GI tract during antiretroviral therapy.

### Fewer Activated Memory CD8^+^ T Cells in the GI Tract during AEI and Posttreatment Correlate with Better Reconstitution Post–Antiretroviral Therapy

Having established that depletion of CD4^+^ T cells (and the CD4:CD8 ratio) in the GI LP persists in the majority of individuals despite therapy, we sought to identify virological and immunological factors at baseline and posttreatment that could account for inter-individual differences. Patients whose CD4^+^ T cell numbers reconstituted to HIV-uninfected levels (Group A, *n* = 9) were compared with patients with persistent CD4^+^ T cell depletion in the LP (Group B, *n* = 21).

With the caveat that patient numbers were relatively limited, no significant differences were noted between groups A and B with respect to baseline viral load, baseline CD4^+^ T cell count, duration of infection prior to biopsy, timing of initiation of antiretroviral therapy, and duration of therapy (Table 2). On comparison of immunological parameters within the GI tract between groups A and B at baseline and posttreatment (Table 3), we noted that the levels of activated memory CD8^+^ T cells were significantly higher at baseline (54.4% ± 17.8%) and posttreatment (29.7% ± 16.2%) in group B patients than in group A patient at baseline (28.4% ± 6.5%, *p* = 0.001) and posttreatment (17.2% ± 8.6%, *p* = 0.01), respectively (Table 3). Effector memory (defined as having a CCR7^−^CD62L^−^ phenotype [25]) cells were compared between groups A and B posttreatment. The levels of CCR7^−^CD62L^−^ CD4^+^ T cells (90.4% ± 5.4%) and CCR7^−^CD62L^−^ CD8^+^ T cells (95.9% ± 2.2%) in group A patients was significantly higher than in group B patients (80.1% ± 12.3% and 91.4% ± 2.5%), respectively (Table 3). Thus, cell counts in the peripheral blood did not correlate with mucosal CD4^+^ T cell reconstitution, whereas local events, such as the levels of activated memory CD8^+^ T cells and effector memory CD4^+^ T cells and CD8^+^ T cells within the GI tract, had a statistically significant correlation.

**Table 2:**
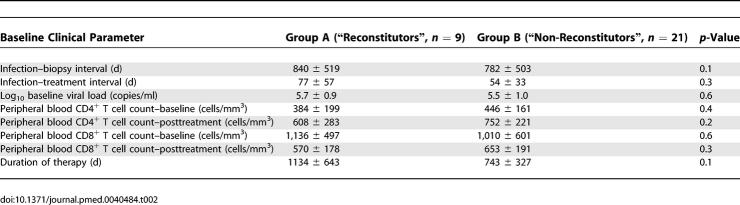
Clinical Comparison between “Reconstitutors” and “Non-Reconstitutors”

**Table 3:**
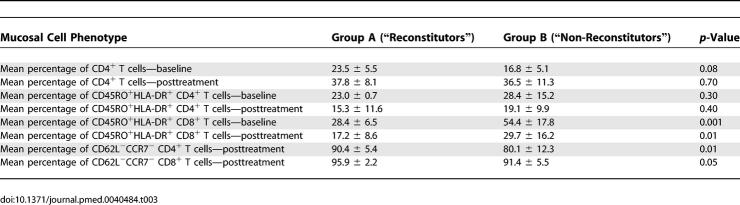
Immunological Comparison between “Reconstitutors” and “Non-Reconstitutors”

### Rare HIV-1 RNA–Expressing Cells Are Detected in Immune-Inductive and Effector Sites Posttreatment

**Figure 5:**
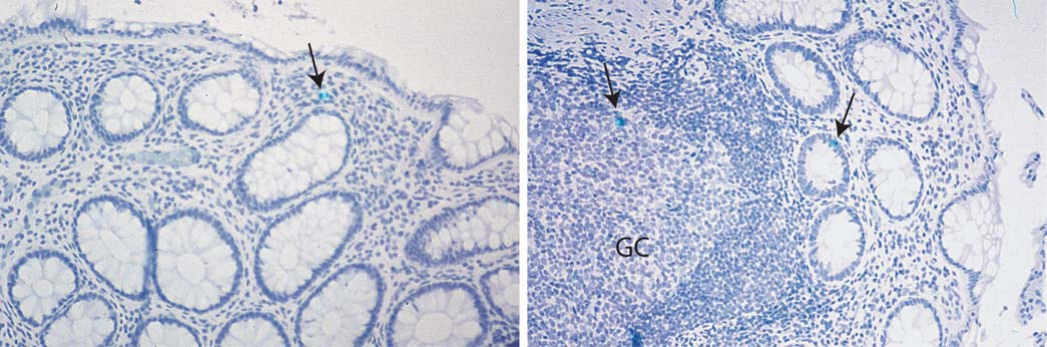
Rare HIV-1 RNA–Expressing Cells Were Detected in the GI Tract during HAART Using a ^35^S-labeled, single-stranded anti-sense RNA probe of HIV-1, in situ hybridization was performed on paraffin-embedded sections as described in Methods. Multiple (4–27) sections per patient were examined. Cells were considered positive for viral gene expression if the grain count was more than six times that of the background. Isolated and rare viral RNA–expressing cells (arrows) were detected in the LP (left panel), T cell zone, and intra-epithelial lymphocytes (right panel), respectively. Original magnification 50×.

GI biopsy sections were hybridized using a radiolabeled probe [18] to detect the presence of viral RNA pre- and posttreatment. RNA-expressing cells were readily detected in immune-inductive sites during AEI [11]. In the HAART-treated patients, rare RNA-expressing cells were detected in immune-inductive and effector sites (Figure 5). Multiple (4–28) biopsy sections had to be analyzed in every case to detect a solitary RNA-expressing cell. At a cellular level, viral RNA expression was visibly less in antiretroviral therapy–treated patients compared with AEI-untreated control participants.

## Discussion

The present study established that in a majority of patients, 70% of our cohort, CD4^+^ T cells do not reconstitute in the LP of the GI tract despite uninterrupted, apparently suppressive, antiretroviral therapy for up to 5–7 y. Based on immunohistochemistry, we estimate that approximately 50%–60% of LP CD4^+^ T cells remain depleted when compared with HIV-uninfected control participants. CD4^+^ T cells expressing the CCR5 receptor or dually expressing the CCR5/CXCR4 receptors are the cell populations most severely affected. Accompanying this persistent lesion are increased levels of activated CD4^+^ and CD8^+^ T cells within the GI tract. In contrast to the degree of CD4^+^ T cell depletion, only rare HIV-1 RNA–expressing cells were detected by in-situ hybridization.

Recent studies of the role of the GI tract have demonstrated that the CD4^+^ T cells residing in the LP are selectively depleted early in the course of acute HIV-1 infection [10–12]. Cross-sectional data on limited numbers of patients suggested that this lesion persists despite the initiation of HAART during acute and early infection [11]. To definitively address this question, we conducted this study of 40 patients who began treatment during AEI, to determine whether this lesion persists during treatment with HAART.

This is, to our knowledge, the largest study describing the effects of antiretroviral therapy initiated during AEI on CD4^+^ T cell reconstitution within the GI tract. Our patients were closely followed, demonstrated excellent adherence to medication regimen, and maintained undetectable plasma viral loads for the study duration. Despite this, a majority of our patients show significant mucosal CD4^+^ T cell depletion. In this regard, our results are in contrast to two prior short-term follow-up studies in HIV-1–infected humans [26] and SIV-infected macaques [27] which inferred that CD4^+^ T cell reconstitution in the GI tract is equivalent to that in peripheral blood.

It is important to recognize that although flow cytometry permits the characterization of cellular phenotype for a large number of cells, the data obtained thus are based on relative cellular percentages, such that an increase in one cell population can affect the percentage of the other. To overcome this potential confounder and to examine absolute numbers of cells, immunohistochemistry was employed. It is noteworthy that, while none of the patients examined by flow cytometry showed “normalization” of CD4^+^ T cell percentage in the GI tract, 30% of the studied patients did show “normalization” of absolute numbers of CD4^+^ T cells within the GI LP. This distinction needs to be taken into consideration so as not to over-interpret suggestions of “a universal lack of mucosal reconstitution.”

Based on the variation in CD4^+^ T cell reconstitution, we sought to understand clinical, immunological, and virological differences between patients. While we did not observe significant clinical differences between the “reconstitutors” and “non-reconstitutors” in terms of baseline plasma viral loads, peripheral blood CD4^+^ T cell count, CD4:CD8 ratio, duration of antiretroviral therapy, or the timing of onset of antiretroviral therapy, we did observe immunological differences within the GI tract: the level of activated memory CD8^+^ T cells at baseline and posttreatment inversely correlated with CD4^+^ T cell reconstitution. In other words, the higher the level of CD8^+^ T cell activation, the less likely that reconstitution would be observed in the GI tract. These data echo the previously observed inverse correlation between levels of activated CD8^+^ T cells, as measured by CD38 expression, and total CD4^+^ T cell counts in the peripheral blood [22–24]. We hypothesize that, owing to viral factors, host factors, or both, the host's inability to down-regulate nonspecific inflammatory responses may be one of the important determinants of the lack of mucosal CD4^+^ T cell reconstitution. Perhaps an even larger study could confirm whether or not correlates of mucosal immune reconstitution exist among clinical parameters such as plasma viral load, duration of therapy, and timing of onset of therapy, among others.

Within the GI tract, there appears to be sub-compartmental variation: immune-inductive sites reconstitute better than immune-effector sites and instead resemble peripheral blood lymphocyte subsets in their reconstitution. It is currently believed that naïve lymphocytes enter inductive sites through high endothelial venules expressing mucosal addressin cell adhesion molecule-1 (MAdCAM-1) interacting with cell surface receptors, particularly α4β7 integrin [28]. Exposure to cognate antigen activates these lymphocytes, and imprinting by resident dendritic cells imparts a homing signal so that, after returning to systemic circulation via the efferent lymphatic vasculature, gut-activated lymphocytes preferentially home to the LP of the gut to execute their effector functions [29,30]. Based on this model, we hypothesize that persistent CD4^+^ T cell depletion within the effector compartment may be due to reductions in the education of gut-homing lymphocytes, to altered cellular recruitment or homing, or to increased cell death due to either ongoing viral replication and/or immune activation in the effector compartment.

Memory cells can be classified on the basis of l-selectin (CD62L) expression into cells with lymphoid tissue–homing potential (CD62L^+^) and non-lymphoid tissue–homing potential (CD62L^−^) [31]. GI lymphocytes fall under the latter category and express high levels of α4β7 integrin [32]. Krzysiek and coworkers found a significant depletion of blood CD4^+^ T cells expressing α4β7 integrin during primary HIV-1 infection and partial recovery following 48 wk of antiretroviral therapy [33]. Our data examined in this context would suggest that lack of cellular recruitment to the GI tract may be one of the factors responsible for persistent mucosal CD4^+^ T cell depletion. Indirect support for our hypothesis was provided by the observation that patients who reconstituted CD4^+^ T cells in the GI tract had a significantly greater percentage of GI effector memory cells (CD62L^−^CCR7^−^) compared with the “non-reconstitutors” (Table 3). These data, when examined in conjunction with recent data from Picker et al. [34], suggest that lack of recruitment of effector memory cells to mucosal sites may be an important determinant of local immune reconstitution. Confirmation of this finding would be based on analysis of specific mucosal homing subsets such as α4β7 integrin—expressing lymphocytes. Such analyses were not possible in the present study because of lack of availability of anti-human α4β7 antibody.

It has been suggested that there may be ongoing viral replication during antiretroviral therapy [35,36]. Persistent depletion of mucosal CD4^+^ T cells expressing CCR5 and CCR5/CXCR4 (Figure 3), the cells that are targeted during acute and early infection, may perhaps be a consequence of HIV-1 associated cytopathicity and/or nonspecific immune activation and apoptosis. Increased levels of activated GI CD4^+^ and CD8^+^ T cells, despite normalization in the peripheral blood (Figure 4A and 4B), could also be indirect evidence of ongoing viral replication at this site. We were able to detect rare cells expressing HIV-1 RNA after hybridization of multiple tissue sections in patients on antiretroviral treatment (Figure 5). However, the presence of isolated viral RNA–expressing cells appears discordant to the degree of CD4^+^ T cell depletion observed. Low sensitivity of in-situ hybridization, owing to the relatively small number of cells that can be examined, may possibly be one of the factors involved. Further studies using PCR-based techniques are underway to address this issue.

The consequences of persistent CD4^+^ T cell depletion in the GI tract are unknown at this point. It is clear that the largest reservoir of immune cells [37] shows a unique profile of CD4^+^ T cell reconstitution during HAART. In the pre-HAART era, opportunistic mucosal infections were a common feature of disease progression. It is evident that, with treatment, patients do not demonstrate any short-term effects of having a 50% loss of CD4^+^ T cells in the GI LP. It is possible that sufficient redundancy in the immune system prevents adverse outcome in chronically HIV-1–infected patients. However, it is important to recognize that, given the projected long-term survival of HIV-1–infected patients, significant loss of mucosal CD4^+^ T cells could accelerate immune senescence with attendant consequences. In recent clinical studies, an increased incidence of polyps and colorectal malignancies has been observed in HIV-1–infected individuals independent of antiretroviral therapy [38,39]. These studies were confined to a single site and involved a homogenous cohort of United States veterans. Whether this finding can be generalized to other groups remains to be determined. Therapeutic use of IL-15 to stimulate the production of effector memory cells with gut-homing potential has recently been demonstrated in a nonhuman primate model [40]. If long-term clinical consequences of persistent mucosal CD4^+^ T cell depletion become evident, modalities such as IL-15 may be considered as adjuncts to HAART in mucosal immune restoration.

While previous studies have focused on the role of the GI tract during AEI, the present study establishes that there is a delay in the majority of patients in mucosal immune reconstitution despite years of apparently successful antiretroviral therapy. Taken together, these data highlight the susceptibility of mucosal sites to HIV-1 and underscore the critical need to develop better mucosal-protection strategies. Recent work in the SIV-macaque model has demonstrated that mucosal delivery of a vaccine may confer better protection against SIV challenge when compared with subcutaneous administration of the same vaccine [41]. Other studies have demonstrated that induction of high-avidity mucosal cytotoxic T lymphocytes by a mucosally delivered vaccine delayed the appearance of plasma virus after rectal SIV challenge in macaques [42]. Similar studies, designed to test the levels of mucosal protection in humans, are clearly needed as better HIV-1 vaccines are developed.

Even though we believe this to be the largest study to date assessing the effect of antiretroviral therapy on the reconstitution of CD4^+^ T cell depletion in the GI tract, there were inherent limitations in a study of this nature. The patient population was selected and not random—particularly those who are willing to undergo repeated biopsy. Furthermore, the group of control participants was limited in size and was not matched to the HIV-1–infected study patients. Our study did not conclusively establish mechanisms of persistent CD4^+^ T cell depletion in the GI tract, and we were therefore unable to definitively identify factors associated with reconstitution—an issue that we believe to be important. It is likely that a larger sample size is needed to address this issue. Finally, the consequences of the persistence of the identified lesion remain obscure. Ongoing research to better understand both the mechanisms of persistent depletion and its implications is in progress.

In summary, we have shown that despite the early initiation of HAART, the marked depletion suffered by the GI mucosal immune system during acute infection does not completely reconstitute in the majority of patients. Though not clinically apparent in the short term, careful observation is warranted as the long-term consequences of this lesion may become evident as the HIV-1–infected population ages.

## Abbreviations

AEI: acute and early HIV-1 infection
EIA: enzyme immunoassay
FITC: fluorescein isothiocyanate
GI: gastrointestinal
HAART: highly active antiretroviral therapy
LP: lamina propria
MAdCAM-1: mucosal addressin cell adhesion molecule-1
MMC: mucosal mononuclear cell
NIH: National Institutes of Health
OD: optical density
OLT: organized lymphoid tissue
PBMC: peripheral blood mononuclear cell
PBS: phosphate-buffered saline
PE: phycoerythrin
PerCP: peridinin chlorophyll-α protein
SIV: simian immunodeficiency virus
