# Correction: Lack of Mucosal Immune Reconstitution during Prolonged Treatment of Acute and Early HIV-1 Infection

**DOI:** 10.1371/journal.pmed.0030546

**Published:** 2006-12-26

**Authors:** Saurabh Mehandru, Michael A Poles, Klara Tenner-Racz, Patrick Jean-Pierre, Victoria Manuelli, Peter Lopez, Anita Shet, Andrea Low, Hiroshi Mohri, Daniel Boden, Paul Racz, Martin Markowitz

In *PLoS Medicine*, volume 3, issue 12: doi:10.1371/journal.pmed.0030484

A related publication should have been cited as a reference in this paper:

Mehandru S, Poles MA, Tenner-Racz K, Manuelli V, Jean-Pierre P, et al. (2006) Mechanisms of gastrointestinal CD4+ T cell depletion during acute and early HIV-1 infection. J Virol. E-pub 25 October 2006

